# Nucleic Acids-Based Biomarkers for Alzheimer’s Disease Diagnosis and Novel Molecules to Treat the Disease

**DOI:** 10.3390/ijms25147893

**Published:** 2024-07-19

**Authors:** Giulia Bivona, Selene Sammataro, Giulio Ghersi

**Affiliations:** 1 Department of Biomedicine, Neurosciences and Advanced Diagnostics, University of Palermo, 90127 Palermo, Italy; 2 Department of Precision Medicine in Medical, Surgical and Critical Care (Me.Pre.C.C.), University of Palermo, 90127 Palermo, Italy; selenesammataro@gmail.com; 3 Department of Biological, Chemical and Pharmaceutical Sciences and Technologies (STEBICEF), University of Palermo, 90128 Palermo, Italy; giulio.ghersi@unipa.it

**Keywords:** Alzheimer’s disease, liquid biopsy, neurodegeneration, extracellular nucleic acids, circulating RNA, extracellular DNA, neuroinflammation

## Abstract

Alzheimer’s disease (AD) represents the most common form of dementia and affects million people worldwide, with a high social burden and considerable economic costs. AD diagnosis benefits from a well-established panel of laboratory tests that allow ruling-in patients, along with FDG and amyloid PET imaging tools. The main laboratory tests used to identify AD patients are Aβ40, Aβ42, the Aβ42/Aβ40 ratio, phosphorylated Tau 181 (pTau181) and total Tau (tTau). Although they are measured preferentially in the cerebrospinal fluid (CSF), some evidence about the possibility for blood-based determination to enter clinical practice is growing up. Unfortunately, CSF biomarkers for AD and, even more, the blood-based ones, present a few flaws, and twenty years of research in this field did not overcome these pitfalls. The tale even worsens when the issue of treating AD is addressed due to the lack of effective strategies despite the many decades of attempts by pharmaceutic industries and scientists. Amyloid-based drugs failed to stop the disease, and no neuroinflammation-based drugs have been demonstrated to work so far. Hence, only symptomatic therapy is available, with no disease-modifying treatment on hand. Such a desolate situation fully justifies the active search for novel biomarkers to be used as reliable tests for AD diagnosis and molecular targets for treating patients. Recently, a novel group of molecules has been identified to be used for AD diagnosis and follow-up, the nuclei acid-based biomarkers. Nucleic acid-based biomarkers are a composite group of extracellular molecules consisting of DNA and RNA alone or in combination with other molecules, including proteins. This review article reports the main findings from the studies carried out on these biomarkers during AD, and highlights their advantages and limitations.

## 1. Introduction

AD is not only a dramatic condition, but it is a lifelong disease as well, spanning 15–25 years from a hidden, symptom-free phase to the apparent one [1]. The diagnosis is based on the combination of clinical and biological criteria, according to the National Institute on Aging and the Alzheimer’s Association Working Group (NIA-AA) guidelines and the Amyloid, Tau and Neurodegeneration ATN framework [1,2,3], respectively. NIA-AA-recommended clinical diagnosis is based on the presence of cognitive and behavioral symptoms and the loss of independence in daily activities, which represents the hallmark of dementia [1]. The ATN scheme-based biological approach incorporates imaging and fluid biomarkers in the diagnostic flowchart, helping identify the disease before overt symptoms occur [3]. Indeed, fluid biomarkers can be detected in cerebrospinal fluid (CSF) in the pre-clinical stage, many years before cognitive alterations develop, and include Aβ, which is characteristic of pre-AD and AD conditions [4]. The complete biomarker panel counts the CSF Aβ40, Aβ42, Aβ42/Aβ40 ratio, pTau 181 and tTau [5,6,7]. CSF still remains the main biological fluid used to measure these markers since their determination in blood displays some limitations (lack of standardization process and quality control program, poor evidence on their clinical usefulness and others) [4,6,8,9,10,11]. A recent review by Leuzy et al. reported blood biomarkers to be reliable tools in clinical practice either for diagnosis or prediction of the disease [12], while highlighting the need for further studies to confirm their validation. However, it has been documented that blood-based biomarkers for AD can be shared among other neurodegenerative disorders, with these molecules showing a relatively less specificity for AD [13]. However, it should be noted that significant flaws also affect CSF biomarkers (Table 1), with consequences on diagnostic, prognostic and therapeutic aspects [8]. Therefore, the search for novel molecules is growing, either to recognize the disease, especially in the pre-clinical phases, or to identify potential targets to set up new approaches to treatment strategies. When considering the importance of reliable biomarkers for disease diagnosis, the impact of these markers in clinical trials should be considered since they also represent useful endpoint measures that help design the study set [14]. Indeed, immunotherapy as a treatment strategy for AD is considered the best attempt so far to slow down its progression and modify the disease course. Monoclonal antibodies binding amyloid species include molecules that are able to attach to the Aβ with different affinity and, albeit modest, some results were achieved through these compounds [15,16].

There has been huge progress in the field of laboratory medicine over time, including the development of advanced technology and the revised understanding and knowledge of disease biomarkers. A new category of biomarkers has been found: the nucleic-acid-based ones. The current article aims to summarize findings from studies focused on novel biomarkers for identifying AD and critically highlights their advantages and limitations.

## 2. Current AD Biomarkers

The main laboratory tests used to identify AD patients are Aβ40, Aβ42, the Aβ42/Aβ40 ratio, phosphorylated Tau 181 (pTau181), phosphorylated Tau 217 (pTau181), phosphorylated Tau 231 (pTau217) and total Tau (tTau). Although they are measured preferentially in the cerebrospinal fluid (CSF), some evidence about the possibility for blood-based determination to enter clinical practice is growing up. Using CSF biomarkers to diagnosis AD display many advantages and represents the gold standard for ruling in AD patients and/or pre-AD mild cognitive impairment (pre-AD MCI) subjects. Also, the ATN scheme takes into account the concentration of these molecules in the CSF of cognitively declined patients to establish the presence of AD dementia. The major advantage of CSF measurement is represented by the standardized procedure and materials used for the determination and the harmonization of the cut-off values obtained among different instrumentation and laboratories worldwide. This alignment is a crucial need for any biomarker to be used effectively in clinical practice and comes from strong efforts over time from researchers and laboratories both in a clinical care setting and research centers. A quality program also guarantees maintaining high-performance analyses [6,7,8]. As for other analytes, the quality program assesses the sample-to-sample variability among different laboratories worldwide regardless of the place, time and instrumentation used, and evaluates the accuracy of the assays and their results in terms of the percentage variation compared to a target value [17]. Particularly, the global control quality program developed by the Alzheimer Association consists of three CSF samples being sent for analysis once a year from the Clinical Neurochemistry Laboratory at the Mölndal campus of the University of Gothenburg, Sweden [18]. Well-established methods and technologies allowed, over time, bypassing analytical issues, including amyloid absorption to plastic tubes, which underestimated the level of Aβ40 and Aβ42 levels in CSF.

Unfortunately, CSF biomarkers for AD present a few flaws, and twenty years of research in this field did not overcome these pitfalls. In 2021, Janelidze et al. compared the performance of eight different Aβ assays for plasma Aβ42/40 in 104 cognitively impaired subjects, demonstrating mass spectrometry (MS) to be superior than the immunoassay methods [19]. Unfortunately, the study was affected by some flaws, including small sample size and the recruitment of different laboratories with possible pre-analytical issues. The same author then analyzed the variability among 10 different pTau isoforms assay methods, documenting MS to be better than other assay methods [20]. However, the analysis was performed on a small sample size and, importantly, the findings cannot be extended to the whole AD patients category, since the patients were not heterogeneous across the AD continuum. The main pitfall of CSF measurement is that the procedure is quite invasive, which leads to another major flaw of these biomarkers. Particularly, the invasive feature of the sample drawing procedure (the lumbar puncture) makes it difficult to repeat serially the determination, which means, in turn, a lack of repeatability. In a chronic, slow disorder like AD, monitoring over time the progression of the disease is crucial, and several determinations of the biomarkers are needed. Hence, the repeatability of that measurement is not a detail, but CSF biomarkers do not display this characteristic. Also, it should be taken into account that only amyloid-related biomarkers benefit from a high specificity, while the alteration of Tau-related molecules seem to be shared among various neurodegenerative diseases and, in general, among the proteinopathies [8].

Regarding the blood-based biomarkers, they include several molecules, ranging from amyloid- and tau-related ones to the microglial ones and those derived from circulating forms of nucleic acids. Namely, plasma biomarkers can be summarized as follows: Aβ42, Aβ40, Aβ42/Aβ40 ratio, pTau181, pTau217, pTau231 (AD classical biomarkers), neuronal-derived exosomes, neuronal-enriched extracellular vesicles (EV), neurofilament light chain, neurogranin, glial acid fibrillary protein (GFAP), triggering receptors expressed in myeloid cells 2 (TREM2) (microglia and neuroinflammation-related biomarker), serum brain-derived neurotrophic factor (BDNF), monocyte chemoattractant protein 1 (MCP1), pro-inflammatory and anti-inflammatory cytokines, extracellular DNA (including nuclear and mitochondrial DNA) and extracellular RNA fragments, including circular, long non-coding and microRNAs. [6,7,8,14,21,22].

However, the most investigated methods so far count the AD classical biomarkers, the Aβ42, Aβ40, Aβ42/Aβ40 ratio, pTau181, pTau217, pTau231 and the neurogranin and neurofilament light chain [7,8]. Despite hard promises on them and great efforts for investigations from various research laboratories, additional studies are needed to demonstrate their utility in clinical practice. Also, a standardization process for plasma measurement is lacking, with no reference materials reagents, instrumentations and cut-off values alignment among diverse laboratories worldwide.

## 3. Nucleic Acid-Based Biomarkers 

Generally, the detection of circulating nucleic acid fragments in blood samples is called liquid biopsy and is successfully used in many neoplastic diseases like inflammatory bowel disease [23,24,25,26]. Nucleic acid-based biomarkers are a composite group of extracellular molecules consisting of DNA and RNA alone or in combination with other molecules, including proteins [23] (Table 2). From a laboratory point of view, the main feature of these extracellular nucleic acid fragments is their presence in several biological fluids, albeit at low concentrations, including blood, CSF, urine and saliva [24]. This is particularly important when considering that quick and easy detection in blood-derived fluids (serum and plasma) would considerably ameliorate laboratory management of AD patients, replacing current time-consuming and invasive procedures required to measure Aβ and pTau-related CSF biomarkers.

Also named cell-free DNA (cfDNA) and cell-free (cfRNA) RNA, the extracellular nucleic acids are released in body fluids upon cellular damage or injury, stimulation, stress and apoptosis [27,28]. For a long time, nucleic acids-based biomarkers have been considered sensor-like molecules, initiating the innate immune response through their interplay with biochemical pathways associated with cellular damage and microbial infections [29]. These sensors, named pattern recognition receptors (PRRs), interact with damage-associated molecular patterns (DAMPs) and pathogen-associated molecular patterns (PAMPs), resulting in the activation of pro-inflammatory signaling pathways. For instance, the transcription factor nuclear factor kappa-B (NF-κB) pathway, which is responsible for pro-inflammatory cytokine production and release, is prompted upon PRRs-DAMPs/PAMPs interplay, thus amplifying inflammation by recruiting other immune cells in tissue ambience [30]. The non-nucleic PAMPs and DAMPs mechanisms in activating the early stage inflammation process throughout the brain are broadly known. In contrast, the cfDNA and cfRNA mechanisms of action are poorly understood, particularly in the central nervous system (CNS) [23]. Besides their DAMPs- and PAMPs-like behavior, cell-free nucleic acids also act as damaging factors themselves. Various cell types have been documented to sustain the pathomechanisms of some diseases, including neurodegenerative, cardiovascular, neoplastic and others [31,32]. Furthermore, some nucleic acids-based compounds are known to promote cell-to-cell communication throughout the brain, which is of major importance when considering that the nervous and immune cells’ interplay regulates many CNS functions [33,34,35]. 

### 3.1. Cell-Free DNA (nuDNA and mtDNA) 

cfDNA species include two main double-stranded DNA extracellular fragment types: nuclear (nuDNA) and mitochondrial (mtDNA). Upon tissue damage, they are released in the extracellular fluids, are detectable in blood and CSF, and then are degraded by DNase enzymes [36]. mt DNA is relatively stable compared to the nuclear one, as it is less susceptible to DNase degradation; hence, it is present in plasma and CSF at higher concentrations [37]. Although normal cfDNA levels in the blood are very low (1–50 ng/mL) [23], in some pathological conditions, including acute brain injury, stroke and autoimmune diseases, they increase considerably [24,38]. cDNA is also included within the neutrophils extracellular traps (NETs), which are aggregates of decondensed chromatin released by neutrophils (and other innate immune cells) upon inflammatory stimuli and also after their adhesion to activated platelets [39]. DNA-containing extracellular traps have been proven to act as protective agents against microbes and prothrombotic factors, along with fibrin [40,41]. Beyond cfDNA, which might serve as a biomarker for AD, the epigenetic state of cfDNA can be evaluated, as well. Particularly, DNA methylation patterns represent one the most investigated epigenetic signatures and provide information about which origin the cfDNA fragment derives from in terms of the damaged tissue and organ [42,43,44].

### 3.2. Cell-Free RNA

Several species of cfRNA are known (Table 2). Ribosomal RNA is the most abundant form in body fluids. It acts indirectly as a DAMP [45,46], stimulating the pro-inflammatory circuit and enhancing pro-inflammatory cytokines synthesis without direct interaction with PRRs [23]. However, some evidence indicates that EV-associated ribosomal RNA can be recognized by TLR3 [47]. It is important to note that TLR3 gene expression is increased in the brains of AD patients and its activation has been found to decrease the disease progression in a mouse model of AD [48,49]. Ribosomal cfRNA can circulate alone or can be associated with proteins and EVs.

MicroRNAs (miRNAs) are small RNA fragments highly expressed in many tissues, including the brain. They are involved in neuroinflammation and neurodegeneration, regulating gene transcription and translation by targeting specific mRNAs [23,24]. miRNAs are considered promoters of cell–cell communication rather than acting as DAMPs [50], and they are often associated with exosomes and EVs. 

Circular RNA (circRNAs) are coding and non-coding RNA fragments, mostly known to represent sponges for miRNAs, hindering their interactions with mRNA and regulating gene expression at a post-transcriptional level [51]. Also, circRNAs can regulate the biological activity of some proteins or be translated themselves [52]. Due to their structural features, circRNAs are not susceptible to degradation and are more stable than the linear species [53].

Long non-coding RNA (lncRNAs) are lengthy RNA species that undergo the processing of proteins that code RNAs [54]. They are responsible for activating or silencing gene expressions due to their association with enzymes modifying chromatin [55]. Further, lncRNAs bind mRNA targets, thus competing with miRNAs and promoting the assembly of translation initiation complexes [56].

tRNA fragments are posit-transcriptional regulators of gene expression and essential mediators of intercellular communication within the CNS [57]. A possible role for tRNAs in driving immune response after neuronal damage during stroke has been [58]. Also, tRNAs have been deemed to lead to neuronal death through glutamate-mediated mechanisms [59].

## 4. Studies on cfDNA and cfRNA in AD

Many studies have explored the concentrations of nucleic acid molecules in blood, plasma, urine and CSF in AD patients, and their possible role in the pathogenesis of the disease has been suggested [18,39,40,41,42,43,44,45,46,47,48,49,50,51,52,60,61,62,63,64,65,66,67,68,69,70,71,72]. The main techniques used to determine extracellular nucleic acids, including miRNA array, high-sensitivity next-generation sequencing (hsNGS), digital PCR and others, display some disadvantages that are common to many laboratory technologies, i.e., the high costs, the need for a standardization process to align procedures and results among laboratories, low specificity and laborious data analyses [73]. Also, it should be noted that their use is mostly limited to neoplasm, with a relatively low application to knowledge in the field of neurodegenerative diseases. The techniques are summarized in Table 3. Also, several analyses have reported significant changes in the plasma or urine levels of cfDNA and cfRNA, and their clinical usefulness as biomarkers of disease have been proposed [10,11,23,24]. However, the following points regarding the possible use of these molecules as biomarkers should be clarified before describing detailed analyses and findings.

Firstly, it should be taken into account that changes in plasma levels of a certain molecule do not allow for the identification of that molecule as a biomarker. Indeed, many laboratory variables typically undergo modification without a clinical significance of their change, which means no clinical course or treatment response can be predicted by measuring the analyte. In this case, the measurement has no usefulness or impact on clinical practice. Clinical usefulness is of enormous relevance for a biomarker, with the unquestionable principle that no biomarker subsists without impacting patient management. Changes in blood or CSF levels of a molecule are very low-impact information due to the lack of significance from any perspective, including the pathogenic, laboratory, clinical and therapeutic ones. 

Another concern is that an alteration of blood levels of a molecule does not mean a link between that molecule and the pathogenesis of a disease, which means that a marker can, though not always, help identify effective treatment strategies by targeting mechanisms underlying the onset and progression of the disease. Considering that some diseases, including AD, have no effective treatment, this aspect is particularly crucial because identifying valuable molecular targets within the AD pathogenic pathways is urgently needed.

Lastly, most of the studies performed on cfDNA and cfRNA levels in AD patient’s blood and CSF display substantial limitations, including limited sample size, high costs, heterogeneity among participants and no specificity for a single neurodegenerative form, meaning that the change in these molecules occurs during many neurological diseases [24]. Also, many studies were performed in in vitro and in vivo models, which represents another limitation in translating the results onto humans.

Given all the above, the main findings from the studies carried out in AD patients to determine cfDNA and cfRNA can be summarized as follows. 

miRNAs are the most studied cell-free nucleic acids measured among AD patients, with miR-202, miR-21-5p, miR126-3p, miR-331-3p, miR-128, miR-433, miR193a-3p, miR-155 and miR133b being the ones analyzed in discrete cohorts (>100 AD patients) [58,74,75,76,77]. Except for Zhang et al. [75], small healthy control groups were recruited, which is poorly justified when considering that the analyses of these studies have been performed on serum samples. Indeed, it is known that recruiting controls for CSF analyses can be difficult, while easy-to-draw samples like blood overcome this problematic aspect of the CSF sample collection. miR-202, miR331-3p, miR-433, miR193a-3p and miR-155 were downregulated in AD patient samples and correlated inversely with cognitive decline [58,74,78]. miR-21-5p, miR126-3p and miR-128 were upregulated in AD samples and positively associated with cognitive impairment [75,76]. Mancuso et al. evaluated mi-223-3p serum levels in AD, Parkinson’s disease (AD) and mild cognitive impairment (MCI) patients and healthy controls. Although the sample size was relatively small (40 AD, 35 MCI, 28 PD, 40 HC), the authors found a lower miR-223-3p concentration in MCI compared to AD and higher miR-223-3p in PD, with a good value of the area under the curve (AUC) within the receiver operating characteristics (ROC) curve analyses evaluating AD and PD patients (0.97) [65]. These findings could be interesting, but it is slightly hazardous to propose miR-223 as a potential biomarker for distinguishing AD and PD based on a ROC curve analysis in less than 100 samples. Robust methods on very large groups, along with a reasonable link between the modification of the molecule and the onset and progression of the disease, are needed to define a molecule as a potential biomarker for the identification of any disease. Also, interventional studies should be performed to demonstrate the efficacy of the biomarker over the existing ones as per the prognosis and treatment response. 

A very different analysis was carried out by Brennan et al., who performed in 2019 a comprehensive evaluation of all the miRNAs’ changes during AD, PD and other neurodegenerative forms. Based on 599 studies on humans evaluating various miRNAs in neurodegenerative diseases, the authors created a knowledge base with the aim of identifying specific and shared miRNAs among different forms of neurodegeneration. The biological samples utilized in the studies were plasma, serum, CSF, peripheral blood mononuclear cells (PBMC) and whole blood, and the core analyses of Brennan’s study were carried out using bioinformatics tools. The importance of Brennan’s study is that such a wide analysis sheds light on those pathways possibly involved in the pathophysiology of the disease, which means helping identify molecular targets for novel treatment approaches [63]. An interesting finding was that the authors demonstrated that hsa-miR-30b-5p was shared among AD, PD and other neurodegenerative forms and displayed a role in the pathogenic mechanisms of these diseases [63]. 

Studies on lncRNAs are relatively few and affected by some flaws, including small study groups. The most relevant analyses (sample size > 100) include those of Zhuang et al. and Chen et al. [78,79]. Zhuang et al. investigated the possible association between the metastasis-associated lung adenocarcinoma transcript-1 loc (lnc-MALAT1) and miR-125b, FOXQ1, PTGS2 and CDK5 with the development and progression of AD. The authors found that plasma MALAT1 was downregulated, while miR- 25b/PTGS2/CDK5 were upregulated in AD plasma and CSF [78]. The analyses were performed on PD patients as well, indicating good discrimination capability of MALAT1 among AD, PD and healthy controls, using a ROC curve analysis. An additional concern, along with the ones affecting miRNA studies, is the role of lnc and miRNAs in predicting AD severity and progression. Indeed, the follow-up period was short (3 years), and patients lost at the follow-up were excluded from the study, which biased the analyses.

Chen et al. evaluated the diagnostic value of lncRNA growth arrest-specific transcript 5 (GAS5) and its relationship with hippocampal volume in Alzheimer’s disease (AD) in plasma samples deriving from 108 AD patients and 83 controls. The authors found that GAS5 is significantly upregulated in AD patients and negatively correlated with the mini-mental state examination (MMSE) score [79]. 

Huang et al. assessed extracellular mtDNA in plasma samples of 90 AD patients, finding lower concentrations than those of 90 controls. The authors suggested that cf-mtDNA is a useful biomarker in neurodegeneration and is related to cellular dysfunction in AD patients [60]. However, they found no correlation between laboratory markers and disease severity, according to the Alzheimer’s Disease Assessment Scale (ADAS). To note is that no mild cognitive decline patients were included in the study, making it difficult to identify prognostic value of these biomarkers. 

Nidadavolu et al. [67] analyzed cfDNA serum levels in 631 subjects in an 8-year follow-up longitudinal study using a digital PCR array. The authors reported cfDNA to be significantly associated with cognitive decline and worsening frailty, and suggested that extracellular DNA fragments can be good predictors of AD development in elderly people. The robust methods and large sample size give strength to Nidadavolu’s study. Lowes et al. [61] determined mtDNA levels in post-mortem ventricular CSF samples from different neurodegenerative diseases, counting AD, PD and Lewy body dementia. The authors reported significantly higher CSF-cfmtDNA levels in patients with dementia but no correlation with some neurodegenerative and mitochondrial markers (neuron-specific enolase, alpha-synuclein, porin, mitochondrial transcription factors and others) [61]. Although an association between elevated CSF-cfmtDNA and the degree of neuropathology, neocortical tau aggregations and dementia have been reported, CSF-cfmtDNA levels in AD samples did not significantly differ from those of matched controls. 

## 5. Biomarkers Identified as Possible Targets for AD Treatment

Monoclonal antibodies against amyloid currently represent the most used approach for AD treatment. Three categories of compounds are known, with patients showing differential affinity for Aβ based on the presentation of amyloid as monomers, dimers and fibrils. The most toxic compound of Aβ are the monomers [80], against which a relatively low affinity is described by the monoclonal antibodies. This could explain the modest clinical results obtained using these molecules in pre-AD and AD patients. Further, it should be taken into account the issue of antibodies synthesis in prokaryotics, affecting all the monoclonal antibodies produced by pharmaceutic industry, rather than in eukaryotic cells. Prokaryotic-derived antibodies processing avoids post-transductional transformations, including glycosylation, which makes the epitopes less immunogenic compared to those produced in eukaryotic cells. This results in a less effective and specific response to these compounds once they are administered and helps explain the modest effectiveness of monoclonal antibodies in some cases [81,82,83].

Beyond amyloid aggregates and accumulation, AD pathogenesis recognizes multiple factors, including proteinopathy and inflammation in the brain. Tentative approaches for treating AD initially were based on limiting the accumulation of amyloid and tau deposits, but no success in slowing down or stopping the disease course has been achieved. Then, targeting molecules involved in the neuroinflammatory process as a valuable strategy to fight AD has been proposed. For a detailed landscape on different molecules developed by biopharmaceuticals industries, the various mechanisms and pathways currently identified as possible targets for treatment and a summary of the several trials currently on going at the time, see the elegant study of Cummings et al. [84].

Regarding one of the most investigated field of analyses, which is neuroinflammation’s role in AD onset and progression, microglia can be considered one of the key players leading to brain inflammation, along with the astrocytes. Hence, biomarkers of microglia are highly investigated. Microglia are immune cells belonging to the myeloid innate arm of immunity, deriving from the yolk sac and colonizing the brain during the early stage of the central nervous system (CNS) development. It has been suggested that, during AD, the deposition of Aβ aggregates within the extracellular space can drive microglia activation which in turn leads to the priming of microglia. The primed phenotype of microglia represents a strong pro-inflammatory subset of these cells, characterized by potent pro-inflammatory feature and neurotoxic behavior. Primed microglia are able to induce hyperphosphorylation of Tau protein and its intracellular deposition [1,85,86,87].

Microglia cells and their markers have been identified as possible targets to develop effective therapeutic products, although using them would be invasive and potentially infeasible. Hence, microglia markers have been mostly searched in blood, with great attention on some receptors expressed on microglia, including the C-X-C motif chemokine Ligand 1 (CX3CL1) receptor and the triggering receptor expressed in myeloid cells 2 (TREM2) [19,60,61,62,63]. Briefly, two main goals represent the focus of research on this topic: (i) modifying baseline features of quiescent microglia in order to reduce the neurotoxic effect of some microglia phenotype (especially the “primed” phenotype); (ii) interfering with the neuron-to-glia communication pathways, which are responsible for maintaining microglia in a quiescent state, in order to avoid both chronic activation of these cells and their conversion toward the primed subset [88,89,90]. In this scenario, some cfRNA, especially tRNA fragments and miRNAs, are known to regulate the communication between neurons and immune cells [23,24] and could be considered potential targets in neuroinflammatory treatment approaches based on the modulation of microglia signals. This comes from the observation that neuron–microglia network and communication prominently aim to keep microglia in a neuroprotective phenotype, with the steady-state, ramified and dark microglia subsets being the most neuroprotective ones [91]. On the other hand, chronic activation and priming of microglia are associated with losing neuroprotection, neuronal damage and death, with the disease-associated and the primed phenotypes being related to AD onset and progression [34,88]. 

Regarding the usefulness of CX3CL1 pathway-related molecules and other neuroinflammatory factors as valuable targets for treatment approaches in AD, some encouraging results have been obtained by [92,93,94], although a few benefits from some cytokines and transcription factors (NF-κB) have been documented [95].

Some concerns still remain around the administration’s route of target molecules and their hazardous synthesis [96] and, importantly, all the findings achieved represent preliminary results. Hence, they should be taken with a grain of salt and require further investigations.

Other biomarkers neuroinflammatory biomarkers that are currently considered for AD therapy include those of inflammasoma complex [97]. However, beyond inflammation in the brain, the major pathways and mechanisms representing novel targets in AD treatment include oxidative stress, energetic metabolism, synaptic plasticity, gut–brain axis and proteinostasis biomarkers [84].

## 6. Conclusions

AD is a devastating disease with poor outcomes for patients and society in terms of treatment and costs. The search for useful biomarkers for AD is an urgent need because they can be used as endpoints in clinical trials. Although current biomarkers are well established to identify the disease, these molecules display some pitfalls, and novel markers are highly investigated, including those linked to the nucleic acids.

Theoretically, free-circulating DNA and RNA represent promising markers to be used for diagnosing and predicting prognosis in AD patients. Also, they could be valuable molecular targets for novel treatment approaches. However, some careful considerations need to be made. Firstly, studies on these markers display many limitations, such as the small sample size, the study set, the subgroup categorization and the methods used. Hence, findings from these studies should be taken with a grain of salt. Furthermore, other concerns include the effect of pre-analytical and analytical conditions that are not fully understood, the role of potential confounders and comorbidities potentially influencing the levels of these biomarkers in biological fluids. 

Although many authors state that cf-DNA and cf-RNA markers are expected to be valuable biomarkers for AD in the future, it seems the path toward this goal is difficult and lengthy.

## Figures and Tables

**Table 1:** Major limitations of CSF and blood biomarkers for AD.

Biomarkers	Flaws
CSF	Invasive; Not repeatable; No easy correlation with disease progression; High-costs; No specificity (pTau and tTau);
Blood	Poor organ specificity (released by organs different from the brain); Analytic issues (binding plasma proteins resulting in biased measurement); Ethnic-based differences among individuals; Lack of harmonization and alignment of materials, procedures and cut-offs

**Table 2:** Major acid nucleic-based biomarkers.

DNA-Based Biomarkers
Nuclear DNA (nuDNA)
Mitochondria DNA (mtDNA)
**RNA-Based Biomarkers**
Circular RNA (circRNA)
Long non-coding RNA (lncRNA)
microRNA (miRNA)
ribosomalRNA (rRNA)
messengerRNA (mRNA)
transferRNA (tRNA)
**Exosomes and Extracellular Vesicles (EVs)**

**Table 3:** Main techniques used to determine nucleic acids levels in body fluid from AD patients.

Techniques	Analyses
miRNA array	miRNA analyses
hsNGS	RNA sequencing
Reduced representation bisulfite sequencing	Methylation profiling
Infinium methylation EPIC bead chip	Methylation profiling
Proximity extension assay (combined immunoassay for Ab recognition + qPCR)	Proteomic analysis
Small input liquid volume extracellular RNA sequencing (SILVER-seq)	RNA sequencing in small volume
Digital PCR	cfDNA analyses
